# Computational modeling and analysis of hippocampal-prefrontal information coding during a spatial decision-making task

**DOI:** 10.3389/fnbeh.2014.00062

**Published:** 2014-03-03

**Authors:** Thomas Jahans-Price, Thomas E. Gorochowski, Matthew A. Wilson, Matthew W. Jones, Rafal Bogacz

**Affiliations:** ^1^School of Physiology and Pharmacology, University of BristolBristol, UK; ^2^Department of Engineering Mathematics, Bristol Centre for Complexity Sciences, University of BristolBristol, UK; ^3^Departments of Brain and Cognitive Sciences and Biology, Picower Institute for Learning and Memory, Massachusetts Institute of TechnologyCambridge, MA, USA; ^4^Department of Computer Science, University of BristolBristol, UK

**Keywords:** hippocampus, prefrontal cortex, decision making, computational modeling, information coding

## Abstract

We introduce a computational model describing rat behavior and the interactions of neural populations processing spatial and mnemonic information during a maze-based, decision-making task. The model integrates sensory input and implements working memory to inform decisions at a choice point, reproducing rat behavioral data and predicting the occurrence of turn- and memory-dependent activity in neuronal networks subserving task performance. We tested these model predictions using a new software toolbox (Maze Query Language, MQL) to analyse activity of medial prefrontal cortical (mPFC) and dorsal hippocampal (dCA1) neurons recorded from six adult rats during task performance. The firing rates of dCA1 neurons discriminated context (i.e., the direction of the previous turn), whilst a subset of mPFC neurons was selective for current turn direction or context, with some conjunctively encoding both. mPFC turn-selective neurons displayed a ramping of activity on approach to the decision turn and turn-selectivity in mPFC was significantly reduced during error trials. These analyses complement data from neurophysiological recordings in non-human primates indicating that firing rates of cortical neurons correlate with integration of sensory evidence used to inform decision-making.

## Introduction

Single cell recordings from frontal and parietal cortical areas during simple choice tasks indicate that the activity levels of neurons in these areas integrate sensory evidence for the available alternatives over time (Kim and Shadlen, 1999; Schall, 2001; Shadlen and Newsome, 2001; Hanks et al., 2012); decisions are made when accumulated activity in these neurons reaches a threshold (Roitman and Shadlen, 2002). This view of decision-making has been formalized in computational models that capture data describing both behavior and neural activity (e.g., Usher and McClelland, 2001; Wang, 2002; Mazurek et al., 2003; Ditterich, 2006; Beck et al., 2008). One such example is the leaky competing accumulator (LCA) model, which uses a set of differential equations to describe the interactions and behavior of populations of neurons during a simple choice between two alternatives (Usher and McClelland, 2001).

**Figure 1:**
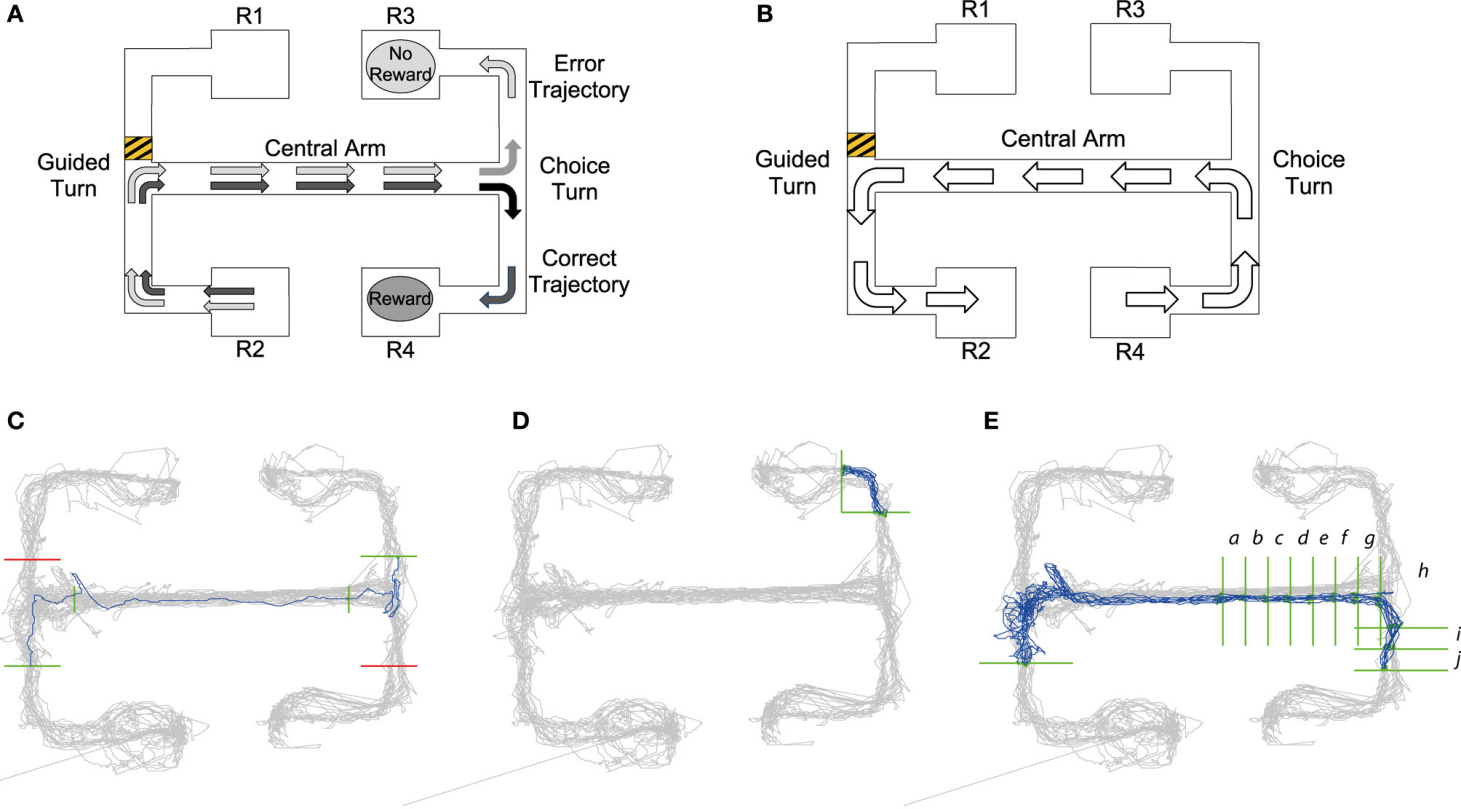
**(A)** Visualization of choice trial starting at the R2 reward point. Black arrows demonstrate the trajectory of a “correct trial” consisting of two turns in the same direction resulting in a reward at reward point R4. Gray arrows show the trajectory of the “error trial” of two different turns resulting in no reward at reward point R3. **(B)** Guided trial starting at R4. The barrier placed in the upper left arm, shows that a trajectory from right to left starting at reward point R4 would result in a guided left turn. Panels **(C–E)**: Queries from MQL toolbox used to select specific trajectories from tracking data: Query lines are shown in green, avoid lines in red, all position data is shown in gray and trajectories satisfying the MQL query are shown in blue. **(C)** Query to return error trials that begin at the lower left reward point. Query lines give intervals for the central arm and choice turn. In MQL query lines are numbered in a listbox and can be selected and amended using the GUI, for details on this process see the MQL website. **(D)** One of the 16 turn queries for the maze. This query returns trajectories from a left turn to the top right reward point. **(E)** A query which selects trajectories of correct trials starting at the lower left reward point, note the additional vertical query lines in the central arm that return the timestamps of intervals a–j leading up to and after the choice turn (see also Figure 4).

Computational approaches like the LCA model have been developed for perceptual choice tasks, but are not directly able to describe maze-based spatial tasks typically used in rodent studies where decisions about turn direction are based on information held in memory. An example of such a spatial decision-making task, and the focus of this paper, is the end-to-end T-maze task illustrated in Figure 1 (Jones and Wilson, 2005a,b). In general, T-maze tasks rely on the integrity of dCA1 and mPFC networks (Wang and Cai, 2006); this particular end-to-end version has been used to study dCA1-mPFC activity and interactions (Jones and Wilson, 2005a,b) and performance is impaired following their pharmacological disruption (Kucewicz et al., 2011). Neurophysiological recordings in rats performing similar tasks corroborate the nature of dCA1-mPFC population activity correlated with spatial decisions (Benchenane et al., 2010; Hyman et al., 2010) and have begun to delineate trial- and task stage-dependent ensemble coding (Baeg et al., 2003). However, the extent to which diverse turn-, route- and memory-dependent firing correlates in rodent mPFC parallel decision-related activity in primate data remains unclear.

To establish a framework for the study of how the brain might control behavior in such maze-based tasks, we develop a minimal model that can successfully perform the above task in simulations. The main benefit of developing the computational model is that it provides a mechanistic description of how neural circuits can control behavior in the task that can be validated in simulations, and generates specific predictions on response patterns of neurons that can be tested in the data. The model is based on the LCA model (Usher and McClelland, 2001) and includes neural populations selective for different choices (i.e., movement patterns) integrating inputs from sensory neurons (indicating position in the maze), but additionally includes neural populations encoding the direction of previous turns. We then analyse data previously recorded during the above task from rat dCA1 and mPFC (Jones and Wilson, 2005a) to investigate whether these brain areas include neurons with response properties corresponding to those of different populations in the model. In order to expedite analyses requiring selection of time series data segments corresponding to specific trajectories or exact regions of a maze, we introduce Maze Query Language (MQL), a generalizable MATLAB software toolbox that enables intuitive querying of experimental behavioral and neurophysiological data via a Graphical User Interface. Using MQL, we analyse the model's predictions of neurons with particular firing rates in specific areas of the maze, demonstrating novel behavioral correlates of mPFC activity likely to contribute to decision-making.

## Materials and methods

### The end-to-end T-maze

The task is schematized in Figure 1 and comprises two main types of trial, which we refer to as “choice trials” and “guided trials.” On a choice trial the rat starts at either R1 or R2 reward points, runs along the connected arm, makes an initial guided turn enforced by the barrier into the central arm, runs along the central arm toward the choice T-junction and must then choose the same turn direction again in order to get a reward. So, as shown in Figure 1A, in trials starting from R2, the rat needs to go to R4 to get rewarded; conversely, on the trials starting from R1 the rat needs to reach R3. On the guided trials (Figure 1B) the rat continues from either R3 or R4 and is guided by barriers back to the starting reward points R1 or R2. The guided trials act as control trials because the rat performs the same running behavior as in the choice trials, but is not required to make any active decisions.

### Computational model

**Figure 2:**
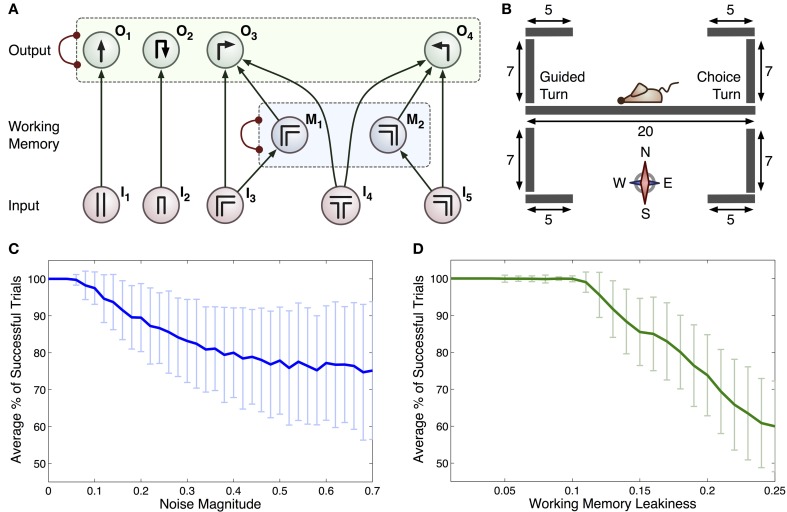
**(A)** Connectionist model for the T-maze task. Arrows represent excitatory connections and arcs ended with circles represent inhibitory connections between all pairs of integrator within a specific group (indicated by dashed rectangles). Each input, denoted *I*_n_, relates to scenarios that the rat may encounter: *I*_1_ straight corridor, *I*_2_ dead end, *I*_3_ right-hand bend, *I*_4_ T-junction, *I*_5_ left-hand bend. Actions that the rat can take are represented by: *O*_1_ move straight ahead, *O*_2_ turn around, *O*_3_ right turn and *O*_4_ left turn. The *M*_1_ and *M*_2_ integrators act as a working memory. **(B)** Geometry of the 2D virtual environment made up of 9 separate line segments. Initial position and orientation of the rat is shown. **(C)** Average % of successful trials with c (standard deviation as overall noise of all integrators) varied. **(D)** Average % of successful trials with standard deviation as leakiness of working memory integrators, k, is varied.

To carry out the decision process for the T-maze task, the model shown in Figure 2A was developed. This is a population level model (McClelland, 1979; Usher and McClelland, 2001) comprising populations of neurons selective for particular aspects of the task (denoted by circles in Figure 2A). The model is structured into three main layers: inputs providing sensory information, working memory to recall previous movements, and outputs defining actions to be taken. The populations in the working memory and output layers were modeled as leaky integrators (i.e., with slowly decreasing excitation in the absence of any input), which accumulated their inputs and additional noise (accounting for random fluctuations in neuronal activity). The activity of inputs to the model was set according to the current position in a simulated maze.

The connections in this model encode the optimal policy for solving the T-maze task. For example, if the rat is in the straight part of the maze it is most likely to go straight: motor output was limited by the constraints of the linear maze, hence turning behavior was only allowed at maze corners or ends (see Figure 2A). All turns were treated equally by the model, regardless of running direction and choice/guided context. The inclusion of a separate working memory component is vital to this task, as the subject must remember the direction of the previous guided turn in order to receive a reward. When the simulated rat is at a turn, sensory input from the population encoding this turn will increase the representation of this turn in the working memory layer and the representation of the other turn will be suppressed due to mutual inhibition between working memory populations. The activity of the working memory populations is used within the model to bias the decision of a left or right hand turn when a T-junction (choice turn) is encountered.

It should be noted that our model of this task describes a learned state, where the connections between populations of neurons are established and static. Other models have been proposed that describe how these connections are developed and how the task is learnt, e.g., by incorporating reinforcement learning to modify cognitive policies (Zilli and Hasselmo, 2008; Lloyd et al., 2012). However, these processes were not the focus of this work.

Actions were performed when excitation of the associated integrator in the output layer exceeded a threshold value and integrator excitations remained fixed while the action took place. This could in some cases lead to the same action being executed multiple times in succession when leakiness was small. These actions were constrained by the maze geometry, ensuring the rat maintained the same position while integrators updated to the new position's stimuli. In addition, as multiple integrators could exceed their threshold at a particular point in time, the integrator with the greatest excitation was chosen. Mutual inhibition was used to establish competition between integrators and lead to the ultimate choice of a single action.

The dynamics for each integrator were described using the following general form:

(1) $$dA=\left({\text{Ext}}_{1}+\ldots +{\text{Ext}}_{n}-kA-w\left({\text{Inh}}_{1}+\ldots +{\text{Inh}}_{m}\right)\right)dt+cdW$$

Where *d*A is the change in integrator excitation, Ext_1_to Ext_*n*_ are the values of excitatory inputs, *k* is the decay rate, *A* is the integrators current excitatory level, *w* is the inhibition rate, Inh_1_ to Inh_*m*_ are the values of inhibitory inputs, *dt* is the time step, and *c* is a scaling factor of the noise *d*W. A Wiener process was used to model noise and ensure appropriate scaling with the time step *dt*. A full model for output and working memory integrators is given by:

(2) $$dO_{1}\;=\left(I_{1}-kO_{1}-w\left(O_{2}+O_{3}+O_{4}\right)\right)dt+cdW,$$

(3) $$dO_{2}\text{ \&}=\text{\&}\left(I_{2}-kO_{2}-w\left(O_{1}+O_{3}+O_{4}\right)\right)dt+cdW,$$

(4) $$dO_{3}\;=\left(I_{3}+I_{4}+M_{1}-kO_{3}-w\left(O_{1}+O_{2}+O_{4}\right)\right)dt+cdW,$$

(5) $$dO_{4}\;=\left(I_{4}+I_{5}+M_{2}-kO_{4}-w\left(O_{1}+O_{2}+O_{3}\right)\right)dt+cdW,$$

(6) $$dM_{1}\;=\left(I_{3}-kM_{1}-wM_{2}\right)dt+cdW,$$

(7) $$dM_{2}\;=\left(I_{5}-kM_{2}-wM_{1}\right)dt+cdW,$$

where *M*_1_ and *M*_2_ are working memory integrators and *I*_1_ to *I*_5_ are external inputs based on the rats current position and orientation within the maze. When *k* = w and *c* = 0, there is no decay of working memories (Bogacz et al., 2006) because under this condition the change in the difference between *M*_1_ and *M*_2_ [that can be obtained by subtracting Equations (6) and (7)] is simply *d*(*M*_1_ − *M*_2_) = (*I*_3_ − I_5_)*dt*. Thus, the change in *M*_1_ − *M*_2_ only depends on inputs, and *M*_1_–*M*_2_ remains constant in their absence.

Sensory inputs were calculated by allowing for this model to drive the behavior of a virtual rat in a simulated T-maze environment. This environment was two-dimensional, consisting of a set of vertical and horizontal line segments over which the rat was able to move. In addition, junctions between these segments were defined, providing information about possible paths through the maze (see Figure 2B for the full maze geometry). We made the simplifying assumptions that the rat has one of four possible orientations (north, east, south or west) and that it moves at a constant speed (2 virtual length units/second) when performing an action. This speed was estimated from recordings of rat movements during experiments and scaled appropriately for the virtual maze. Variation in trial durations (for example during error trials) was accommodated by the virtual rat pausing during the choice process, i.e., while the activity of all accumulators in the output layer was below the threshold. If the rat attempted to move outside a line segment (e.g., into a wall), the position remained unchanged. On the basis of current position, line segment and orientation, one of the inputs (*I*_1_, *I*_2_, *I*_3_, *I*_4_, *I*_5_) was set to 1, while others were set to 0.

Unless stated otherwise, simulations used the parameter values *w* = 0.2, *k* = 0.2, *c* = 0.0001, and decision threshold was set to 1. To account for statistical variation in simulations, 300 duplicates of 1000 time units were executed for the parameter set tested in Figure 2. In all cases the rat was initially placed in the center of the maze facing west toward the guided turn (see Figure 2B) and all model variables were initialized to *O_i_* = *M_i_* = 0. Equations (2)–(7) were solved using the Euler method with *dt* = 0.1, and if any of the model variables became negative after an integration step, their value was reset to 0. The performance of the animals was characterized by a fraction of correct trials, so this single statistic was not sufficient to constrain multiple parameters of the model. Therefore, we used a sample combination of parameter values for which the model was able to recapitulate the range of behavioral performance observed across animals.

### Experimental data

We re-analyse data recorded by Jones and Wilson (2005a,b). dCA1 pyramidal cell layer (−3.6 mm, +2.2 mm from bregma) and deep-layer prelimbic cortical (mPFC +3.2 mm, +0.6 mm) action potential spike times were recorded from multiple tetrode electrodes (implanted under isoflurane anaesthesia) in 6 male Long-Evans rats running the end-to-end T-maze, alongside video-tracked head position sampled at 30 Hz. Rats were food restricted (to 85% of free-feeding weight) and trained in the task until consistent >85% performance prior to tetrode implantation; reward was chocolate-flavored milk drink. Data from an entire single recording session at least 7 days post-surgery from each rat are analyzed here; sessions lasted 18–35 min per rat (average 21 min) and include spike times from a total of 77 dCA1 place cells and 78 putative pyramidal neurons in mPFC, recorded during a total of 16 error trials and 77 correct trials. The average task success rate during these sessions was 83 ± 5% (s.e.m). See Jones and Wilson (2005a,b) for complete details.

### Selecting trials

The experimental data were analyzed using MQL, a new software toolbox for analysing time series data recorded during maze-based tasks. The toolbox aids analysis by correcting for loss of positional signal from video camera tracking of rats (described in detail by Jahans-Price, 2010) and flexibly selecting a subset of recorded trajectories on the basis of user specified constraints called “queries.” The MQL toolbox is written in MATLAB and is downloadable with additional documentation, screenshots and tutorials, from http://www.cs.bris.ac.uk/Research/MachineLearning/mql/

Queries created in MQL consist of a number of constraints and return the subset of position data (and associated spike-times) which meet all of them. Each constraint is a horizontal or vertical “query line” that intersects with part of the maze. The query lines specify a desired trajectory, as MQL only selects trajectories which intersect the query lines in a specified order. Namely, when the query is run, MQL collects series of chronological position data which cross all the query lines in the order they are defined, i.e., the line defined by query line 1 must be crossed first. Additional “avoid” queries can also be added to fine-tune trajectory selection (shown in red in Figures 1C,E).

Figure 1C illustrates how queries are constructed to return a specific trajectory, e.g., retrieving all error choice trials (see Section The End-to-End T-maze for details) starting from R2 that make an incorrect left turn down to reward point R3. The first query line, intersecting with the lower left arm, ensures that trajectories start in that arm, i.e., from R2. The second and third query lines in the central arm make sure the trajectory passes through the central arm. The fourth and final query line ensures the run is an incorrect trial, finishing at R3. Query lines 3 and 4 are precisely placed to give the timestamps for the beginning and end of the choice turn. MQL uses query lines to export the timestamps at which they are crossed, providing data to calculate timings and firing rates occurring between query lines.

Using timestamps from queries like that shown in Figure 1C, it was possible to compare timing and neural activity on correct vs. error trials for the run along the central arm and during the choice turn. Jones and Wilson (2005a,b) define the central arm as the central three-quarters of the arm. So as to define the central arm in the same way, vertical query lines were placed one eighth into the arm at both ends giving timestamps for the central three-quarters. We define the choice turn as the final eighth of the central arm and the same distance into either the upper or lower arm. Therefore, horizontal query lines were placed in the upper and lower arms the same distance from the center of the T-junction as the vertical query line defining the end of the central arm.

Our model's prediction of turn-selective neural activity on the maze was tested by analysing activity from individual neurons during all the turns. In order to achieve this MQL was used to create 16 queries for each maze, a left turn and right turn query for each of the possible 8 turns in the maze. Each of these queries returned the start and the finish of each trajectory during a particular turn allowing the calculation of the firing rates of all neurons during this turn. A sample query is shown in Figure 1D. Query lines into and out of the central arm used the same query line placement defined by the central arm and choice turn, turns into or out of reward points used the same x-coordinate as the central arm query lines where possible. However, due to different rats following different trajectories whether entering or exiting the reward point, query lines had to be adjusted in order to fully capture the turn.

To examine the activity of turn selective neurons leading up to and during the choice turn queries similar to those used for analysis of the choice turn were used. Again four queries that select the four types of choice trial were used (example of one trial shown in Figure 1E). In order to analyse firing rates as the choice turn was approached along the central arm addition vertical query lines were added. This created a number of intervals approximately 6 cm in length along the central arm at which timestamps could be obtained.

The validity of position data is calculated by MQL, this information can be used to exclude position data which is potentially inaccurate (See website http://www.cs.bris.acwww.cs.bris.ac.uk/Research/MachineLearning/mql/ for code and method). For the analysis of the firing rate or the time between two query lines, we used trajectories in which both points of intersection with the query lines are valid. When for example analysing the firing rate of neurons during the choice turn (Figure 1C), only the timestamps for entering and exiting the turn need to be accurate. Validity at query line also allows other areas in the maze to have signal loss if they are not required for the current analysis.

### D-prime analysis

To investigate how much information neurons encode about the direction of a turn, we used d-prime analysis as a measure of how accurately two conditions could be discriminated based on a neuron's firing rate. This approach assumes the distributions of firing rates across trials in two conditions *a* and *b* are approximately Gaussian and have the same variance σ^2^, but differing means μ_*a*_ and μ_*b*_. The d-prime value gives the distance between the means and therefore the separation between the distributions (Dayan and Abbott, 2001).

(8) $$d\prime =\frac{\mu_{a}-\mu_{b}}{\sigma }$$

The larger the d-prime value, the higher the discriminability between these two distributions and the greater the selectivity of a neuron for one condition over the other.

We first used d-prime analysis to investigate how well individual neurons discriminated between left and right turns, using MQL queries (Figure 1D) to select the timestamps for the two turn directions. We calculated the average firing rates for neurons during each of these trajectories returned by MQL, giving left and right turn distributions of firing rates for each neuron (see Figure 5 for examples of such distributions for two neurons). We then computed the means of the left turn and right turn firing rate distributions for each neuron. To calculate the common variance we subtracted the mean from each distribution and combined them. Substituting these means and variance into Equation (8), we obtained a d-prime measure of turn selectivity for all analyzed neurons.

Secondly, we used an analogous approach to determine how well central arm firing rates of individual neurons discriminated between trials originating from reward point R1 and reward point R2.

In order for neurons to be included in both d-prime analyses they were required to demonstrate either turn selectivity or trajectory selectivity. Neurons that passed a two factor analysis of variance: left-turn activity vs. right-turn activity with *p* < 0.05 were categorized as turn selective. Neurons that passed a *t*-test comparing firing rates in the central arm on trials from R1 vs. from R2 (*p* < 0.05) were categorized as trajectory selective.

## Results

### Computational model

Simulations were run to test if the computational model summarized in Figure 2A could generate similar behavior to that seen under experimental conditions. Without noise, the model was able to make correct choices on all trials, whereas rats showed an average accuracy of 83 ± 5%. We therefore added a noise component by setting *c* > 0, introducing errors in the form of unavoidable fluctuations in neural activity and enabling a more accurate simulation of experimental behavior. We observed that the accuracy of the model could be reduced in two ways: (i) by increasing the level of noise (Figure 2C), or (ii) by increasing the leakiness of working memory [*k* in Equations (6)–(7)] and thus reducing the ability for information to be retained for long periods of time (Figure 2D).

### Turn selective neurons

**Figure 3:**
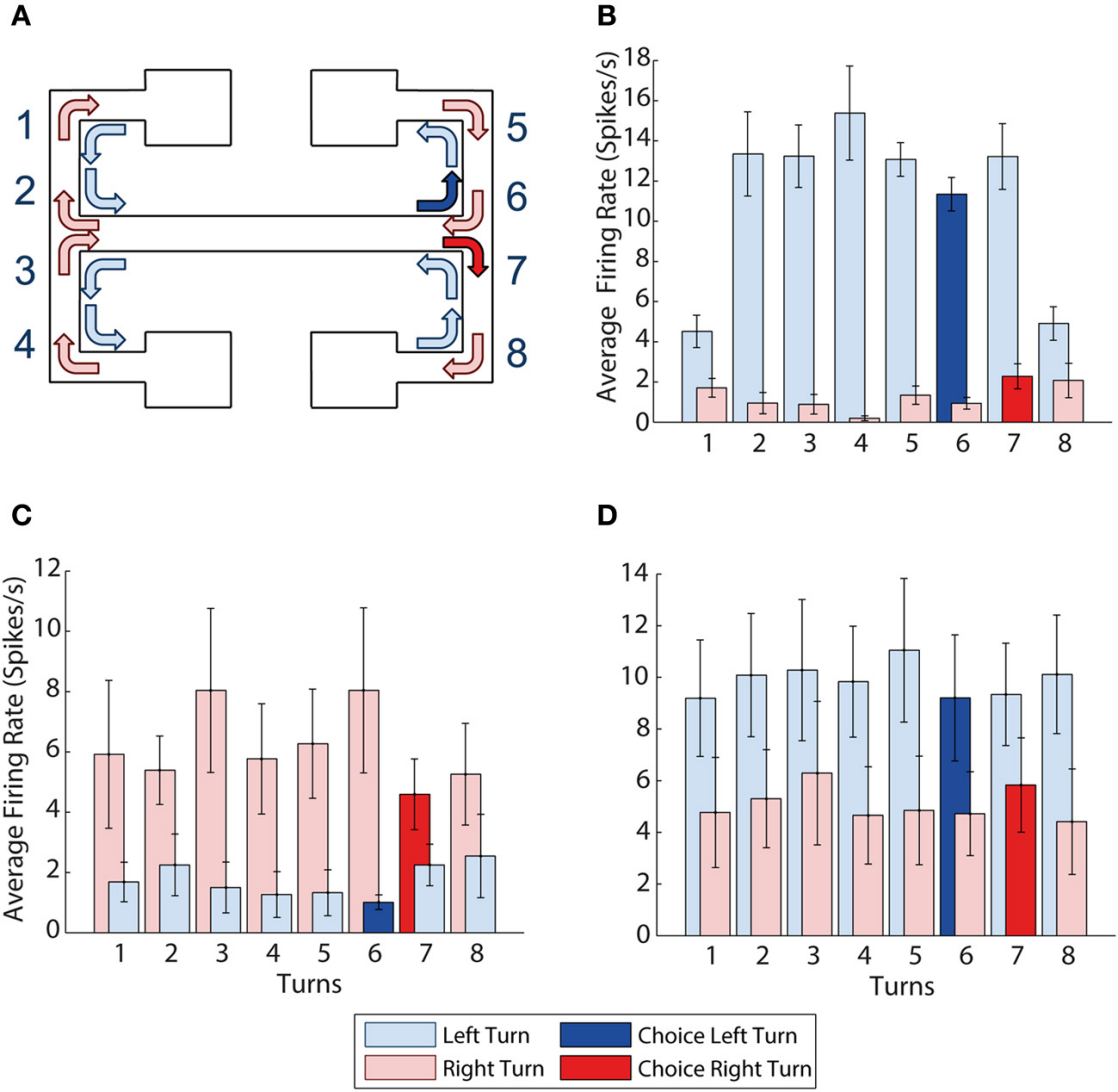
**mPFC activity is turn dependent. (A)** Visualization of all the left and right turns in the maze, left turns shown with light blue arrows, right turns shown with light red arrows, choice right turn shown in solid red, choice left turn shown in solid blue. **(B)** Example of a neuron with activity selective for left turns. It shows higher average firing rates during all 8 left turns than during all 8 right turns. **(C,D)** Turn selectivity does not appear to be a result of one turn being over represented **(C)** The average activity at each of the turns for all 9 right turn preferring neurons, turn 7 is the choice turn. **(D)** The average activity at each of the turn for all 10 left turn preferring neurons, turn 6 is the choice turn. Error bars show the standard error of the mean.

Our model predicts that in the spatial task we should expect to find turn-selective neurons (shown in the output layer of the model) with firing rates significantly different on left vs. right turns. To test for this, we compared activity during the 8 left and 8 right turns of the maze (Figure 3A) for every neuron using a two factor analysis of variance. A neuron was considered to be turn selective if there was a significant effect of the direction factor (*p* < 0.05). We did not demand a minimum firing rate of a neuron for inclusion in the turn selectivity analysis, as two factor analysis of variance requires a sufficient firing rate during at least one turn direction in order to support significance.

Figure 3B shows a sample neuron selective for left turns, firing at a consistently higher rate than during right turns (*p* = 0.0005). We found a total of 19 prefrontal neurons (24% of the population recorded) selective for a particular turn direction. 10 of the 19 turn neurons were left- and 9 right-turn selective. In what follows we refer to the turn direction associated with higher firing rates as “preferred turn” and the opposite direction as the “unpreferred turn.” CA1 neurons were not turn-selective by this measure, although some neurons did show limited selectivity on a subset of the 8 possible turns.

As the prefrontal cortex is suggested to encode the learning of rules and strategies that guide behavior (e.g., Wallis et al., 2001) it is possible that the firing rate differences of turn neurons could be driven by differential activity around a single turn that is particularly important to the task, e.g., the choice turn. For example, if firing rates during the choice turns were preferentially influencing the previous analyses, we would expect to see higher average firing rates for left and right turn-preferring neurons on exit from the central arm during choice runs (at corners 6 and 7 on Figure 3A respectively). However, Figures 3C,D illustrate that average firing rates over all 19 turn-selective neurons are similar across all 8 turns during both the preferred and unpreferred turn directions.

### Prospective mPFC activity during approaches to the choice turn

Our computational model suggests that turn-selective neurons are influenced by working memory neurons, and as part of the decision-making process should therefore increase their firing rates prospectively before a decision to ensure a turn in their preferred direction is made. Conversely, due to mutual inhibition between the neuron populations representing alternative actions (Figure 2A), we would expect that neurons selective for the unpreferred turn should also decrease their firing rates.

**Figure 4:**
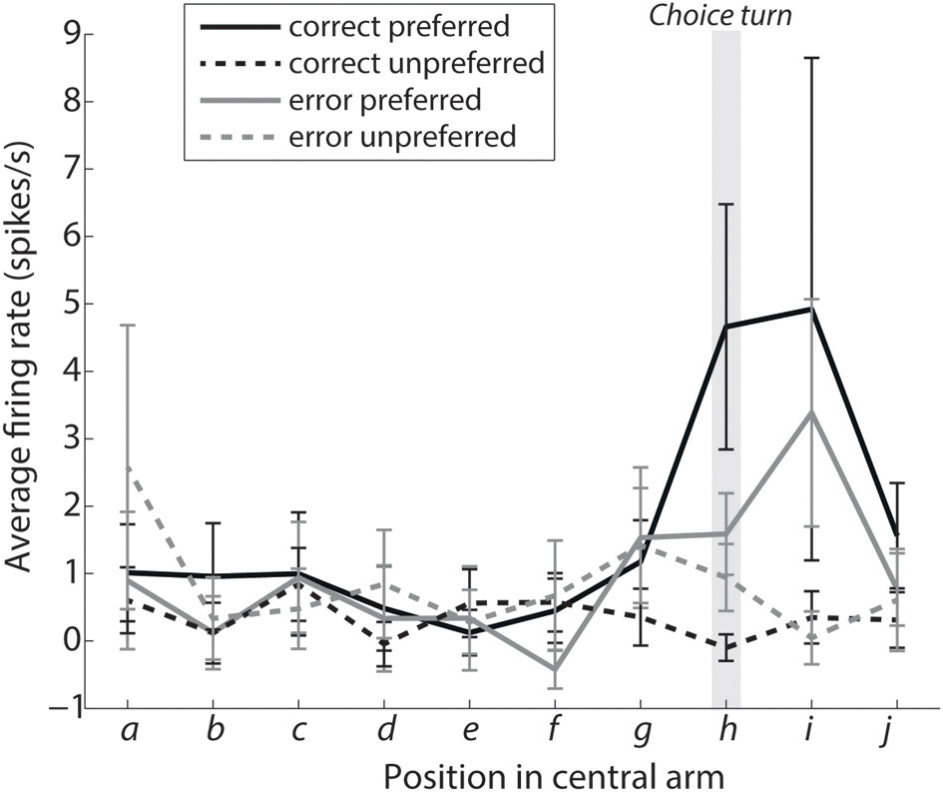
**Turn selective mPFC neurons display prospective ramping of activity before a choice turn in their preferred turn direction.** The curves show the average firing rates during intervals leading to the choice turn labeled as shown in Figure 1E. Interval h encompasses the choice turn *per se*, with intervals i and j directly following the choice turn. Solid lines show the average activity of turn-selective neurons from trials when the rat turned in direction preferred by the neuron, while dashed lines are based on trials when the rat turned in the opposite direction. Please note that selectivity for the preferred direction during the choice turn is significantly reduced during error trials (shown in gray, *P* < 0.05).

Figure 4 shows the average activity of turn selective neurons as the rat approaches the choice turn. Each position on the Figure 4 x-axis represents from left to right the intervals between query lines in Figure 1E, with position h being the interval at which the rat makes the choice turn. The activity value gives the average firing rate of all 19 turn-selective neurons calculated from their individual average activity during the given interval. The four lines plotted on Figure 4 represent the average firing rates during the intervals for the four types of choice trial (two correct trials and two error trials). The preferred and unpreferred terms define the direction of the choice turn and whether it is the direction a turn-selective neuron is associated with. Specifically, a correct preferred trial for a right turn selective neuron is a choice trial starting from the bottom left reward point (R2) with a right choice turn, while for a left turn selective neuron it is during a trial starting at the top left reward point (R1) involving a left choice turn. Using the preferred and unpreferred terms allowed us to combine the firing rates from both sets of turn-selective neurons.

Figure 4 shows a prospective ramping in activity of turn-selective neurons during the “preferred correct” condition and reduction of activity during the “unpreferred correct” condition. This is consistent with turn-selective neurons representing future turn direction and inhibiting the alternative choice. The same effect can be seen for the error conditions although in a reduced manner. A two-factor analysis of variance was used to compare the conditions preferred vs. unpreferred and correct vs. error. The turn-selective neurons showed a significant selectivity for preferred vs. unpreferred during the choice turn (*p* = 0.0083; position h). This selectivity was reduced during error trials (factor: interaction, *p* = 0.0424).

### Conjunctive coding: are turn- and trajectory-selective neurons separate populations?

In order to make a correct choice, information about the past trajectory needs to be maintained while the rat is in the central arm until the choice point is reached. For all neurons recorded, we investigated whether their firing rates in the central arm were significantly different depending on whether the trajectory was initiated from R1 or R2. We found 12 such “trajectory-selective neurons,” 8 in mPFC and 4 in dCA1.

Our model, for conceptual simplicity, assumes that turn-selective and trajectory-selective neurons (in the working memory layer) constitute separate populations. However, we now investigate whether the mPFC populations of turn- and trajectory-selective neurons revealed by the above analyses are in fact separate or overlapping, with some neurons conjunctively coding for both turn and trajectory. We used a method of signal detection called d-prime (see Methods) to calculate the selectivity of individual neurons for a given condition.

**Figure 5:**
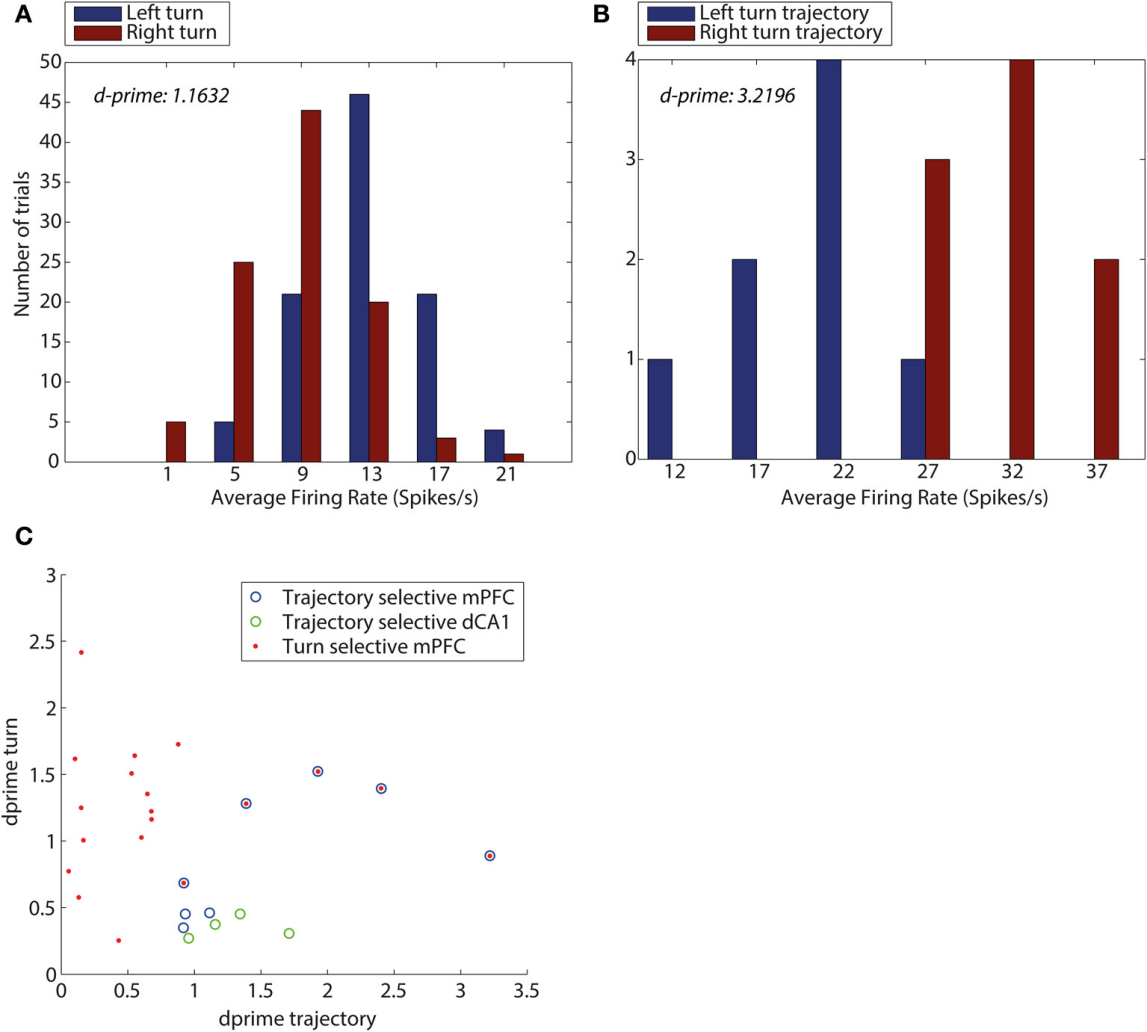
**D-prime values for turn direction and trajectory reveal an overlap between mPFC neurons encoding turn and trajectory. (A)** D-prime score for a mPFC turn selective neuron, measuring separation of distributions of average firing rates (spikes/s), during left turns (blue) and right turns (red). **(B)** D-prime score for a mPFC trajectory selective neuron during trajectories making a left turn into the central arm (blue) and trajectories making a right turn into the central arm (red). D-prime values of 1.1632 and 3.2196 (left to right), plots correspond to two mPFC neurons shown in panel **(C)**. **(C)** D-prime values for mPFC and dCA1 neurons measuring selectivity for turn direction and maze trajectory. Each symbol corresponds to one neuron, its y-coordinate is equal to the d-prime measuring the difference in firing rate distributions during turns left and right, the x-coordinate is equal to d-prime measure of difference in firing rates during the central arm, between trajectories from top and bottom of the maze. Neurons identified as turn selective are shown in red (mPFC), neurons identified as trajectory selective shown in blue (mPFC) and green (HPC), Neurons which were identified as both turn and trajectory selective are shown as blue with red in the center.

Figure 5 illustrates that dCA1 contained neurons that were exclusively trajectory-selective with high d-prime values only present for trajectory (Figure 5C). In contrast, the mPFC contained an array of neuron types with 3 showing trajectory selectivity, 14 showing turn selectivity, and 5 mPFC neurons showing selectivity for the conjunction of turn and trajectory with high d-prime in both cases.

## Discussion

The analysis in the present manuscript extends the original analyses of the data made by Jones and Wilson (2005a) in several ways. Jones and Wilson reported trial-dependent activity in mPFC on the central arm of the maze, and here we additionally examine neural activity at corners. We identified prefrontal neurons selective for turn direction during a spatial task and observed a prospective ramping of activity for the preferred direction before a decision. During error trials we saw a reduction in the selectivity for turn direction suggesting a lower confidence in the decision. We found that the mPFC turn-selective neurons overlapped with a population of trajectory-selective (working memory) neurons, with neurons from both populations encoding both concepts.

### Computational model

For conceptual simplicity, our computational model assumed separate neural populations selective for turns direction and previous trajectory. By contrast, our analyses revealed neurons selective for both. To include this experimental observation, one could develop a model inspired by that of Machens et al. (2005) which uses mutual inhibition to implement both decision making and working memory as a single state variable. This model demonstrates that by using non-linear dynamics, simple modules of neurons are able to change the configuration of their dynamical properties and Machens et al. (2005) propose this as a possible explanation for the frontal lobes ability to switch between different rules quickly. Alternatively, one could develop a model on a different scale, i.e., including description of individual neurons, rather than populations. This would be an interesting direction for future work. In our model it is not clear specifically where errors in the task result from. They could be explained by a failure either in the working memory layer or in the output layer. Errors in our model resulting from increased noise or working memory leak both fit the behavioral data but make different predictions; working memory leak predicts that the working memory neurons should be less selective on error trials. We found no significant difference in the selectivity between error and correct trials, however the number of working memory neurons we found did not provide sufficient statistical power to establish if these neurons decrease their selectivity on errors. This would be an interesting question to address in the future with a larger dataset.

### Turn selective neurons

We are not aware of any previous reports of prefrontal turn selective neurons in the rat mPFC, although neurons selective for parts of a maze associated with particular behaviors (Jung et al., 1998) and neurons with contextual dependent activity (Hyman et al., 2012) have been observed. Similar turn-selectivity has been reported in other regions known to process spatial information, including posterior parietal cortex (Whitlock et al., 2008; Nitz, 2009). One hypothesis explaining the presence of mPFC turn selective neurons is that combinations of turns are important for success in the T-maze. Therefore, the ability to remember the turns made everywhere in the maze is part of a strategy that develops over time as the rat learns the task. As all data were collected from rats already trained in the T-maze task an experience-dependent analysis examining the evolution of turn-selectivity was not possible here.

### Ramping activity of turn-selective neurons

Analysis of mPFC turn-selective neuron activity during preferred trials showed an increase before and during the choice turn. Similar increases in firing rate have been observed by Ramus and Eichenbaum (2000) and Narayanan and Laubach (2009), who found similar ramping firing rates in rats during an odor-matching task and reaction time task respectively. It is also consistent with Baeg et al. (2003) where an elevation in firing rate of prefrontal neurons is found in rats during a working memory task. Ramping of mPFC firing rates on the central arm of the T-maze overlaps with regions of the maze where CA1-mPFC theta coherence peaks (Jones and Wilson, 2005a,b), and may reflect integration of spatial information into working memory and/or decision-making. Recently, a gradual increase in firing rate has been demonstrated in posterior parietal cortex in a task where a rat was required to make a decision on the basis of information it integrated from auditory stimuli (Hanks et al., 2012). Gradual build-up of activity before a decision observed in rats is similar to the experimental results of Shadlen and Newsome (2001) who demonstrated that neurons in lateral intraparietal area of monkey integrate sensory information during decision making tasks.

The firing rate of turn-selective neurons during the correct trials also appears to support our computational model. Specifically, during the correct trials unpreferred neurons did not increase their firing rates, but instead appeared to show lateral inhibition from the preferred neurons leading to significant differences in their activity during the choice turn. This is an example in the model of evidence favoring one choice being greater and thus resulting in neurons representing the other choice becoming inhibited, making the turn neurons highly selective for the correct choice. Consistent with a role of mPFC inhibitory mechanisms, periods of turn-selective mPFC firing coincide with periods of augmented CA1-mPFC interactions, which have in turn been linked to enhanced interneuron-pyramidal cell coordination on during similar spatial tasks (Benchenane et al., 2010). On error trials the difference in activity between the two groups of neurons is not significant and the turn neurons are significantly less selective than during correct trials. This is a potential explanation for errors consistent with the model as similar activity on preferred and unpreferred error trials indicates a much closer competition between the two choices and similar levels of evidence for each choice. When the choice is more difficult, according to the model evidence is integrated more slowly with one choice taking over and inhibiting the other. This is demonstrated firstly with error trials taking longer on average (Median error trials 2.08 s, median correct 1.06 s, Wilcoxon rank sum *p* = 0.02132), and secondly by results present in Figure 4. Figure 4 shows the gray lines representing preferred and unpreferred neurons, both increase their firing rate, but while the solid gray line continues to increase the dashed gray slows and eventually drops during the choice turn indicating increased inhibition from the opposing turn-selective neurons.

The difference in selectivity when comparing correct and error trials is, however, in contrast with the findings of Roitman and Shadlen (2002). Their experiment showed that in a decision making task, similar levels of activity were recorded during correct and error trials from neurons in the parietal cortex. They saw a consistent decision threshold that was reached by both correct and error trials. However, Kiani and Shadlen (2009) showed a reduction in selectivity for low confidence decisions, which is consistent with our findings. It is also worth noting that the precise point at which the decision is made is more difficult to define in the T-maze task compared to Roitman and Shadlen (2002), which involved an eye movement to make a decision. Additionally, in our analysis we group the neural activity by position on the maze rather than by time before the decision is made. It could therefore be possible that we simply are not seeing the common decision threshold reached due to imprecise time aligning of the trials.

### Conjunctive coding

From the turn-selective neurons identified in mPFC, 5 were also shown to be trajectory selective. This is likely a product of the way data is encoded in the mPFC, whereby neurons are used to encode multiple different concepts (Wallis et al., 2001). Hence, neurons responsible for remembering the direction of the last turn can also be selective for the direction of the current turn. Furthermore, finding groups of neurons in the mPFC selective for turns, trajectory and both these functions, fits with the idea that the mPFC codes for individual components of a task and also the conjunction (Wallis et al., 2001; Rigotti et al., 2013). Conjunctive coding can provide computational advantages to a randomly connected neural network encoding a number of task rules. As the number of rules is increases, the neural network can scale linearly in size if the neurons are able to respond to a large proportion of events (Rigotti et al., 2010). Conjunctive coding is also suggested play a role in the flexibility of the prefrontal cortex facilitating quick adaption to execute new tasks (Rigotti et al., 2013)

### Future work

It would be interesting to replicate some of the analyses here for a variation of the task that included variable difficulty (as in the study of Hanks et al., 2012). This would generate more error trials and allow us to test a prediction of our model, that the rate at which turn selective mPFC neuronal activity ramps would slow as task difficultly increases. Recording from the prefrontal cortex while the T-maze task is being learnt by rats would enable us to test whether turn-selective neurons develop over time as hypothesized. With an increased dataset that included more working memory neurons, it could be possible to test model predictions regarding causes of errors. Namely, with additional working memory neurons we could examine whether they exhibit a reduction in selectivity during error trials, implicating the leak parameter in creating errors. It would be also interesting to study a modified version of the task in which the rule determining which direction the rats should take at the choice point switches thorough experiment (as considered by Lloyd et al., 2012). Recording of neural activity in this task would enable us to examine the role information coding plays in regard to prefrontal flexibility (Miller and Cohen, 2001) and switches in behavioral strategy (Rigotti et al., 2013). Posterior parietal cortex and prefrontal cortex receive spatial input from the hippocampus and now turn direction selective neurons have been observed in both regions. Recording in these areas together would allow us to compare information coding and observe any correlates between these regions.

### Conclusions

This study demonstrates that neurons in mPFC can encode individual concepts or the conjunction of multiple concepts, as we show that mPFC cells encode either turn direction, maze trajectory or both of these task aspects. Information about turn direction is important for completion of our task and is represented in mPFC by turn selective cells. These turn selective cells show activity suggesting they may correspond to the accumulators in our computational model and integrate the evidence before making the choice. This suggests that we may be able to study decision making processes in rodents, in a similar way as previously studied in non-human primates.

### Conflict of interest statement

The authors declare that the research was conducted in the absence of any commercial or financial relationships that could be construed as a potential conflict of interest.
